# Biocuration with insufficient resources and fixed timelines

**DOI:** 10.1093/database/bav116

**Published:** 2015-12-23

**Authors:** Raul Rodriguez-Esteban

**Affiliations:** ^1^Roche Pharmaceutical Research and Early Development, pRED Informatics, Roche Innovation Center Basel, Basel 4070, Switzerland

## Abstract

Biological curation, or biocuration, is often studied from the perspective of creating and maintaining databases that have the goal of mapping and tracking certain areas of biology. However, much biocuration is, in fact, dedicated to finite and time-limited projects in which insufficient resources demand trade-offs. This typically more ephemeral type of curation is nonetheless of importance in biomedical research. Here, I propose a framework to understand such restricted curation projects from the point of view of return on curation (ROC), value, efficiency and productivity. Moreover, I suggest general strategies to optimize these curation efforts, such as the ‘multiple strategies’ approach, as well as a metric called overhead that can be used in the context of managing curation resources.

## Introduction

Biocuration is the enterprise of annotating, organizing and displaying biological data by humans (1). Its focus is on tasks for which automatic means cannot achieve the same level of quality as trained human curators. Due to the costly nature of employing trained curators, an important topic of biocuration research has been to increase the efficiency of curatorial work. Proposals have covered such topics as semi-automated workflows (2–7), user-friendly curation interfaces (8–12), improved annotation (13, 14), crowdsourcing (15, 16), outsourcing (17) and sharing of resources across curation efforts (18).

Biocuration is often analyzed in the context of continually maintained biological databases that have the goal of following a growing area of biology. For example, the database BioGRID aims to curate all genetic and protein interactions from the literature about *S. cerevisiae* and *S. pombe* (19), and the focus of the model organism database (MOD) Wormbase is to curate all genetic and molecular data published about *C. elegans* (20). There are also databases whose goals are more ambitious than the resources they have available and thus prioritize their curation efforts. The MOD Ecocyc aims to curate, among other things, functional information for all *E. coli* K-12 genes and proteins (21). However, Ecocyc must prioritize its curation efforts due to the high publication rate for its model organism. Another example is BindingDB, a database of protein–ligand binding affinities, which selects proteins of special importance to focus its curation efforts (22).

Such biological databases are ‘open’ curation projects that work with the implicit or explicit premise of an unrestricted timeline, which is necessary to accomplish the goal of following a scientific field. This premise, however, does not apply to humbler curation projects with fixed timelines and limited resources. In such projects, not all relevant data may be curated and, due to their more transient or contingent nature, good-enough and best-effort results can be acceptable. Pharmaceutical drug projects, for example, prioritize efforts when multiple sources of data with different degree of importance are available. In general, many biological experiments are undertaken only after limited data curation because analyzing every piece of existing information is not crucial.

More broadly speaking, there is a need to understand biocuration in a formal way that acknowledges that there are resource constraints involved. Biocuration studies only use concepts such as curation speed, curation rate and full-time equivalent curator time (23–25). On the other hand, many existing information retrieval concepts do not fully apply to biocuration because, as for other biological applications (26), settings differ from those of typical information retrieval problems.

## Results

In a curation project, there is a set of items to be curated *x = {x_i_}*, with *i = 1…n*, so that *|x| = n*. Every item *x_i_* in this set takes a certain amount of time *T_i_* to be curated. The average curation time is therefore:

$$\bar{T}\;=\;\frac{\sum_{i=1...n}\;T_{i}}{n},$$

which can be often estimated by curating a subset of items or from past experience in similar curation projects. The average curation speed is the inverse of the average curation time, *v* = 1/*T*. The value of *v* is a function of many factors, such as the number of curators employed and the layers of quality control involved. The trade-off between quality and quantity in curation, though important, is not further considered here. The maximum amount of curation time available and average speed, in the cases discussed, is a fixed quantity per project. Together, *v* and *T_max_* represent the curation resources available. Curation projects with an unrestricted timeline do not have a *T_max_*, or the *T_max_* is not a fixed value, and the curation resources available are defined mainly by *v*.

Curation projects with fixed timelines and sufficient resources are characterized by *v · T_max _ ≥* *__n*. Of interest here, however, are curation projects for which the curation of all items is not possible, so that *v · T_max_* *__<* *__n*. In that sense, these are curation projects with ‘fixed, insufficient’ resources. One way to deal with such projects is to use a filtering strategy *f* on the initial items *x^f ^=^ ^f(x)*, where *x^f^ ⊂ x*, that can reduce the number of items to be curated to *|x^f^| = m < n* so that *v · T_max_* *__≥* *__m*. To simplify, our focus initially is on the particular case that all curation resources available end up being used, meaning that *v · T_max_* *__=* *__m*.

## Value, return on curation and productivity

Because items are often of different value (‘not all data are created equal’), it is generally of interest that the set of items that will be curated *x^f^* be of the highest value possible in order to increase the return on curation (ROC). For that, the aim of the filtering strategy *f* should be to include as many valuable items belonging to *x* into *x^f^* as possible. Choosing higher value items from the initial set *x* can be in some cases straightforward. However, the value of the items may not be fully known until they are curated. There, the filtering strategy can be based, instead, on an estimation of the value. Such estimations are typical of document *triaging*, which is an established task in the realm of text mining (27–29) that involves the ranking of documents according to their curation priority.

A binary scale is a simple way to measure an item’s value: an item is either valuable or not. Using a binary scale, well-known information retrieval concepts can be easily translated and applied. Items from *x^f^* that are valuable can be called true positives (TPs) and items that are not valuable, false positives (FPs). False negatives (FNs) are the valuable items in *x* that are not present in *x^f^*, while true negatives (TNs) are the non-valuable items in *x* that are not present in *x^f^*. The aim of the filtering strategy in such a scenario is to maximize the density of TPs in *x^f^*, which can be assessed using the precision metric: *TP/(TP + FP)*. Precision is, thus, related to the curation productivity: the higher the precision the more TPs per unit of time that can be curated. If TPs and FPs take the same average amount of time to be curated, then the production rate of TPs can be defined as *v_TP_* *=* *__v ·* precision. *v_TP_* is a measure of curation productivity.

Precision has some shortcomings as a metric when used for curation. For one, it cannot be easily used to compare the *efficiency* of filtering strategies. For example, imagine three filtering strategies *f_75%_*, *f_50%_* and *f_25%_* that produce an output *x^f^* of identical size but of precision 75, 50 and 25%, respectively. While these filtering strategies are equally spaced by a precision difference of 25%, the number of FPs produced by *f_50%_* is 100% higher than the number produced by *f_75%_*, while the number of FPs produced by *f_25%_* is 50% higher than the number produced by *f_50%_*. Thus, the difference in the number of FPs is not linearly proportional to the difference in precision. Similarly, precision cannot be easily used to compare filtering strategies in their ability to produce a fixed number of TPs. To produce 100 TPs, the *f_75%_* strategy produces 33 wasteful FPs, while *f_50%_* produces 100 FPs and *f_25%_* produces 300 FPs.

These issues with precision also apply to other metrics derived from precision, such as the F-measure, which is further discussed below. Beyond that, the notion of precision as used in information retrieval is not easily expanded into the realm of curation resources. For example, existing differences in the amount of time that takes to curate FPs and TPs cannot be introduced in the precision metric without twisting its general meaning.

## Overhead and efficiency

A metric that can be used to evaluate the results of filtering strategies is overhead (O), which I define in its simplest form as the ratio of FPs to TPs:

$$O\;=\;\frac{FP}{TP}.$$

In this simple form, overhead is related to precision in the following way:

$$Precision\;=\;\frac{1}{1+O}.$$

The ratio of FPs to TPs is sometimes used when evaluating diagnostic tests and screenings, as an alternative to the positive predictive value (which is equivalent to precision) (e.g. (30, 31)). In that context, it is used to indicate how many patients are wrongly diagnosed per patient that is correctly diagnosed.

In the context of curation, overhead is the additional curation effort required due to the presence of FPs in *x^f^*. Overhead, thus, reflects the ‘wasted’ curation time spent on curating FPs (the sum of all FPs is also known as overgeneration) that could be better used curating TPs instead. Therefore, overhead is an efficiency measure. For example, the overhead for *f_75%_*, *f_50%_* and *f_25%_* is 33, 100 and 300%, respectively, which more appropriately depicts the differences in wasted effort involved in curating these three sets.

The overhead metric shows that there is little gain in curation efficiency with filtering strategies of a precision value above a certain level, say > 80%. The overhead corresponding to a precision of 85% is 18%, while the overhead corresponding to a precision of 75% is 33%. Thus, a 10% increase in precision leads only to a 15% decrease in overhead. Therefore, improving precision brings diminishing returns and, beyond a certain level, may not be worth the effort.

**Figure 1:**
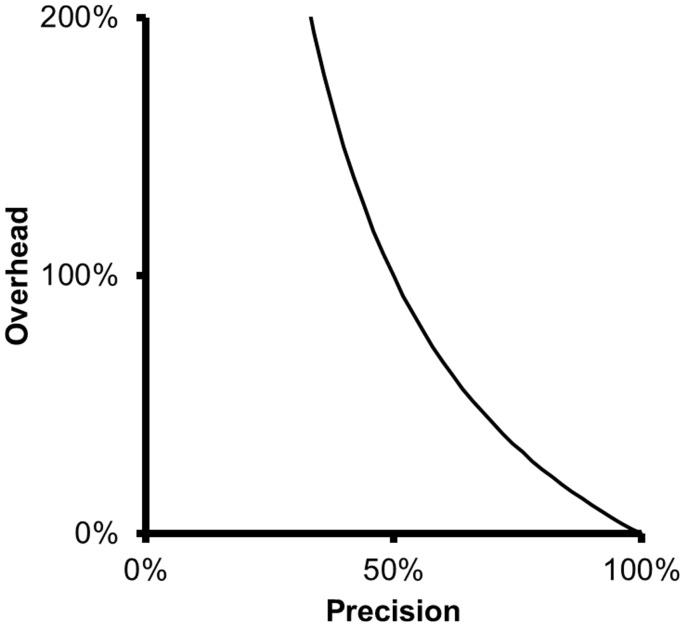
Relationship between precision and overhead. As precision decreases, overhead grows quickly and inversely to precision.

At low precision levels, on the other hand, small changes in precision can have large impact in the overhead, as can be seen in Figure 1. A 40% precision is associated to an overhead of 150%, while a 50% precision is associated with a 100% overhead. Thus, in this case, a 10% increase in precision leads to a 50% decrease in overhead.

A virtue of the concept of overhead is that it can be easily transplanted to different curation settings. In cases for which curating FPs does not take the same amount of time as curating TPs, overhead can be weighed by a factor. For example, if the average curation time for an FP is ${\bar{T}}_{FP}$ and for a TP is ${\bar{T}}_{TP}$, then this factor can be expressed as ${\bar{T}}_{FP}/{\bar{T}}_{TP}$ and overhead as

$$O\;=\;\frac{FP\cdot {\bar{T}}_{FP}}{TP\cdot {\bar{T}}_{TP}}.$$

The rate of production of TPs (*v_TP_*) expressed in TPs curated per unit of time can be then expressed as:

$$v_{TP}\;=\;\frac{1}{{\bar{T}}_{TP}(1+O)}$$

Thus, overhead relates the average time that it takes to produce a TP in an ideal scenario in which there are no FPs (overhead of 0%), with the average time it takes to produce a TP when FPs are actually present. The larger the overhead, the larger the difference between these two values.

## Value scales

The discussion until this point has been concerned with a binary value scale. Value, however, can be highly nuanced depending on the curation goals (32). The value of an item can be based on the experimental conditions in which it was measured, its novelty, or the impact factor of the journal where it was published (33). An appropriate value scale could take any cardinality or even be continuous. Generically, if the value of a curated item *x_i_* is *w_i_*, then the goal of the filtering strategy could be defined as maximizing the combined value of the elements in the set *x^f^*, or $\sum w^{f}$. One typical, straightforward strategy to maximize $\sum w^{f}$ is to rank items by estimated value and to exclude items that rank below a certain cut-off. In such cases, the cut-off point may not need to be established beforehand if items are curated in descending order starting with the item of highest value and until time reaches *T_max_*. This strategy has the virtue of eliminating the need to estimate the curation speed *v*.

The overhead metric with non-binary value scales could be defined in a weighted manner:

$$O\;=\;\frac{\sum_{i}(w_{max}-w_{i}^{f})}{\sum_{i}(w_{i}^{f}-w_{min})},$$

for all *w^f^_i_ ∈ w^f^*, *w^f^_i_ ∈ [w_min,_ w_max_]*. In this formulation, overhead is a measure linked to the ‘creation of value,’ which is another way to look at the ROC. It relates to the potential value that could be created curating the most valuable items but is wasted instead by curating items of value inferior to the maximum, *w_max_*. This formulation can be further refined for cases in which curating elements of different value *w_i_* takes, on average, a different amount of curation time *T(w_i_)*:

$$O\;=\;\frac{\sum_{i}(w_{max}-w_{i}^{f})T(w_{i}^{f})}{\sum_{i}(w_{i}^{f}-w_{min})T(w_{i}^{f})}.$$

In the simplest case *w_min _=_ _w_FP_ = 0*, *w_max _=_ _w_TP_ = 1*, *w_i_ ∈ {w_FP_, w_TP_}*, *T(w_min_) = T(w_max_)* and overhead becomes the ratio of FPs to TPs.

As it has been mentioned, productivity and efficiency metrics can be created before (prospectively) or after curation. If measured before curation, the values of the items and the average curation times expected can be estimations instead of the actual quantities. It is worth noting that the value of an item may also change over time once it has been curated. Thus, filtering strategies may not look optimal retrospectively and any metrics that have been utilized to design them, such as precision or overhead, inaccurate. To take that into account, value estimations could be considered with a confidence interval.

## Redundancy and novelty

A complicating factor in assigning value to an item is due to its multiplicity or redundancy, which might not be readily apparent before curation. A similar or identical item may have been curated in a previous curation project or appear more than once within the same curation project. Given that a curation project might involve several curators, an item might be in fact unique for more than one curator independently, especially if the curation is not coordinated in a centralized manner (for example, by having central curation records that are updated continuously).

Previous studies (34–36) have shown that redundant items or items that lack novelty might be of little interest for many practical purposes in biomedical research. However, this depends on the curation project because items that are similar but stem from independent experimentation can possess value due to their confirmatory nature. To adjust for each situation, metrics such as precision or overhead can be re-defined on the basis of uniquely curated items or by using novelty as a factor in the assignation of value. Redundant or already curated items can be assigned, e.g. the same value that is assigned to an FP.

## Double filtering criteria

An even more complicated scenario arises if the items to be curated have to be valued according to more than one criterion: a multi-dimensional problem, of which the simplest case is the two-dimensional. Here, I will mention one typical setting that involves two dimensions: (1) the estimated value of an item, and (2) the estimated probability that an item is a TP. These are both estimations of value but are of a different nature; one involving the properties of the item and the other involving the quality of the filtering strategy. Such a double-ranking has been tentatively studied for extraction of interactions from text, using a high-value gold-standard as a training set (37). Besides this example, another approach is to consider the value of FPs to be the minimum *w_min_*, smaller than any other possible value, *w_min_* *__≤* *__w_i_.* If we then consider *p(x_i_)* the probability of an item *x_i_* to be a TP, then the overhead metric can be adjusted to be:

$$O\;=\;\frac{\begin{array}{c} \left\{\sum (w_{max}-w_{i}^{f})T(w_{i}^{f})p(x_{i}^{f})+\sum (w_{max}-w_{min})T(w_{i}^{f})(1-p(x_{i}^{f})\right\} \end{array}}{\sum (w_{i}^{f}-w_{min})T(w_{i}^{f})p(x_{i}^{f})},$$

in which values, probabilities and average times are all estimations.

## Adjusting filtering strategies

Available curation resources can differ from project to project depending on the circumstances. For example, the curation speed *v* can change based on the number of curators employed. The maximum curation time available *T_max_* can vary from project to project due to stricter or more lenient deadlines. Therefore the creation of the set *x^f^* needs to be a function of the curation resources available for a given project so that it is ensured that *v · T_max_* *≥* *|x^f^|* *=* *m*.

One way to attain this flexibility is to possess several filtering strategies *f* that are able to produce sets of different size *m*. I call this approach the ‘multiple strategies’ approach. It involves adopting the best possible filtering strategies and choosing the most adequate for each specific curation project. This is like acquiring a screwdriver set, with one screwdriver for each type of screw.

To build a set of filtering strategies it is necessary to use adequate metrics to select them. Swet proposed that an ideal measure for retrieval should be a single number (26, 38). Thus, evaluations of information retrieval systems rely on single metrics such as area under the curve (AUC), F-measure (39) and TAP-k (26). However, to pick the best filtering strategies one metric is not enough. Two filtering strategies *f_i_* and *f_j_* can produce sets with the same precision or overhead but different size *m_i_* and *m_j_*. Instead, a complementary information retrieval metric such as recall (in the binary case), which is a function of the number of TPs and FNs (TP/(TP  +  FN)), can be used in addition to precision or overhead to compare filtering strategies. For example, if the recall of *f_i_* is higher than that of *f_j_*, it means that *m_i_* *>* *__m_j_*.

One additional issue when selecting the best filtering strategies is that each strategy may be associated with a different cost in terms of resources required to apply it. Such costs may affect, for example, the *T_max_* available and thus influence the choice of strategy.

**Figure 2:**
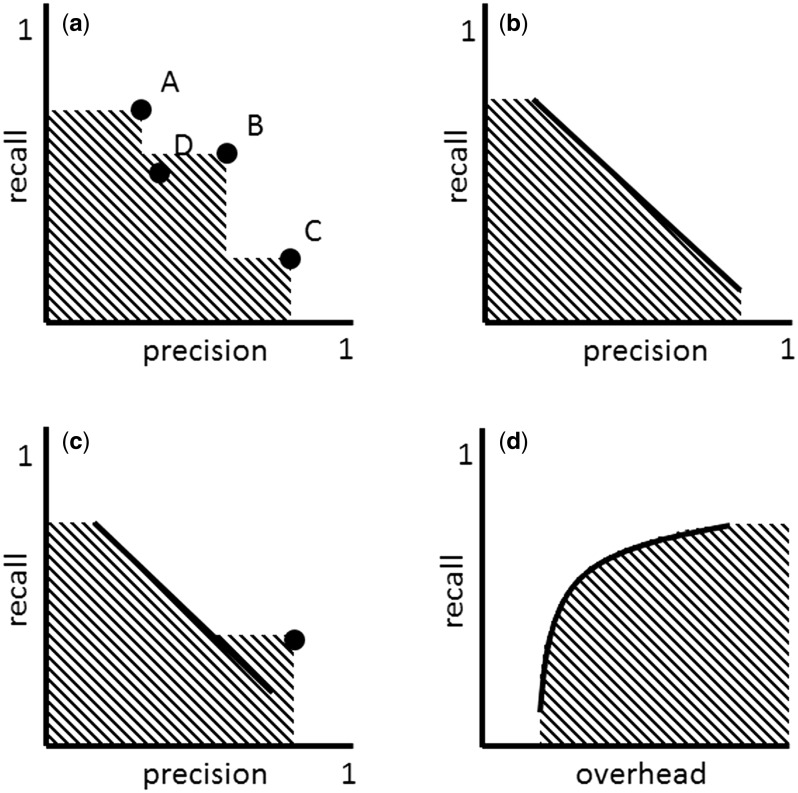
(**a**) ‘Multiple strategies’ approach in the precision-recall space. Strategy A can take the role of ‘high recall’ strategy, while C that of ‘high precision’ and B that of ‘compromise.’ A new strategy D is inferior to the set of strategies A, B and C, because it falls into the area covered (AC) by these strategies. (**b**) Adjustable strategy. (**c**) ‘Multiple strategies’ approach involving an adjustable strategy (defined by the line) and a non-adjustable strategy (defined by the dot). (**d**) Adjustable strategy in the overhead-recall space.

The multiple strategies approach can involve strategies of very different nature, such as ‘high precision,’ ‘high recall’ and ‘compromise’ strategies. Figure 2a shows a set of such strategies mapped to the precision-recall space. These strategies define an area (shaded area) that is conceptually very similar to that of area under the curve (AUC) and which I call area covered (AC).

The AC can grow with the addition of new strategies. As can be seen in Figure 2a, strategy C increases the AC defined by strategies A and B. Strategy D, on the other hand, doesn’t increase the AC defined by strategies A, B and C, because it is an inferior strategy, in particular inferior to strategy B. Any new strategy that falls within the AC is inferior to the set of existing strategies A, B and C. The goal of improving a set of multiple strategies involves increasing the AC by introducing new strategies that are superior or that complement existing strategies. In practice, this can be done through benchmarking exercises. Rebholz-Schuhmann *et al.* (41), e.g. benchmarked gene name taggers of very different nature. In the results of that study, it can be seen that each tagger has different precision-recall properties and therefore can be used in different filtering strategies. A selected set of gene name taggers with different precision-recall properties could be put together to create an effective multiple strategy to identify gene names in text.

Defining, creating and maintaining multiple strategies can be costly. An alternative or complementary strategy is to use filtering strategies with a recall (or precision/overhead) that can be adjusted (Figure 2b and c). By increasing the recall of an adjustable filtering strategy *f* the size of the resulting dataset *x^f^* can grow, which translates into the addition of more TPs and FPs into *x^f^*. Typically for adjustable filtering strategies, when recall is increased, precision decreases (42) and, therefore, overhead increases. Thus, the precision-recall function is monotonically decreasing, while the overhead-recall function is monotonically increasing (Figure 2b and d).

## F-measure

The well-known F-measure, which is the harmonic mean of precision and recall, deserves further examination. It was made popular by its use at the 4th Message Understanding Conference (MUC), a competition organized to improve the state of the art in information retrieval (40). It was chosen because it summarizes precision and recall in one measure, and because it favors strategies that balance precision and recall. For example, a tool with precision 90% and recall 10% has an F-measure of only 18%, while a tool with precision 50% and recall 50% has an F-measure of 50%. The F-measure penalizes, thus, the difference between precision and recall, and the larger the difference the higher the penalty.

Nonetheless, a ‘high precision’ strategy with 90% precision and 10% recall may be suitable for a curation project when curation resources are scarce, regardless of its low F-measure. The F-measure is, in fact a simplification of the original *F*_β_ measure,

$$F_{\beta }\;=\;\frac{(\beta^{2}+1)PR}{\beta^{2}P+R},$$

which was designed to deal with different precision/recall scenarios. The F-measure is a particular case of the *F*_β_ measure in which β  =  1. Values of β  <  1 are used in cases in which precision is favored and values of β  >  1 for cases in which recall is favored.

A problem that the F-measure inherits from the precision metric is the difficulty of comparing the efficiency of filtering strategies using only the F-measure. Differences in F-measure for two filtering strategies are more or less significant depending on the absolute value of the F-measures and not just on their difference. From the point of view of curation, two filtering strategies with F-measure of 95 and 90%, respectively, are more similar than two filtering strategies with F-measure of 25 and 20%. (This can be easily observed at break-even point, i.e. when F-measure =  recall = precision.) This is because as the values of precision and recall increase, the F-measure decelerates its growth (the second derivate of the F-measure is negative for the range of all possible values of precision and recall). Thus, two metrics, such as precision or overhead together with recall, are a better guide to comparing filtering strategies than one metric such as the F-measure alone.

## Saving resources

Thus far we have been assuming that all curation resources available are used, *v* · *T_max_* *__=* *__m*. However, in practice, there might be multiple projects with different priorities competing for resources. Resource saving in one curation project can lead to more resources being available for other projects. One approach to resource allocation across curation projects is to avoid wasting time on low-value items using an absolute threshold of a minimum estimated value *w_min_* acceptable for an item. Thus, no item to be curated would have an estimated value below *w_min_*. With such a threshold it could become possible that no single curation project surpasses the maximum resources allowed for an individual project or that the sum of the resources needed for all curation projects does not surpass the maximum amount of resources available. This threshold can be defined in advance in absolute terms, as *E*-values are used for sequence alignment results (*E*-values are used to assess the statistical significance of a sequence alignment in algorithms such as BLAST). An alternative to this approach would be to optimize the value of time allowed $T_{max}^{j}$ for every project *j* so that the sum of overheads *O_j_* for all projects is minimized: $min\sum_{j}O_{j}$, with $\sum_{j}T_{max}^{j}\;=\;T_{max}$ This is an optimization problem that can be approached using any of the overhead measures proposed.

## Use case

As an example, I describe a use case involving the curation of relations between proteins from the literature. In particular, the focus of the use case is on the curation of relations between the human proteins interleukin 32 (IL32), which is a pro-inflammatory cytokine, and the anti-inflammatory cytokine interleukin 10 (IL10) in Medline abstracts up to 2014.

As baseline, 27 abstracts mention IL32 and IL10 (or synonyms) in Medline publications up to 2014. To reduce the work load of curating 27 abstracts, a ‘high recall’ filtering strategy based on co-occurrence can be applied to select abstracts in which IL10 and IL32 are mentioned at least once together in the same sentence. (This can be done with the help of the software Linguamatics I2E (43).) Such strategy yields 18 abstracts, of which 11 contain sentences that relate, even if tenuously, IL10 and IL32. Thus the overhead in this curation project is, to a first approximation, 7/18  = 39%. In other words, FPs represent a 39% of additional effort.

However, this overhead value is somewhat misleading because, in this case, curating FPs requires less effort than curating TPs. An FP can be decided after reading the text, while a TP requires further storing and annotation. Thus, if the average time to curate a $\text{TP(}{\bar{T}}_{TP})$ is 2 times larger than the average time it takes to curate an FP then the overhead can be computed as:

$$O\;=\;\frac{FP\cdot {\bar{T}}_{FP}}{TP\cdot {\bar{T}}_{TP}}\;=\;\frac{7}{18\times 2}\;=19\%$$

where 19% is the additional curation time due to the FPs. In order to reduce this curation load further, we can prioritize the results by filtering for sentences that, besides IL10 and IL32, mention also interaction-related keywords (43, 44). Thus, this would assign these sentences a higher probability of signaling TPs than those sentences without these keywords. This filtering, however, only reduces the number of results to 17, eliminating one of the FPs and reducing the overhead slightly. Nonetheless, we could be interested in zooming in on those sentences with certain interaction keywords that signal more potential value, such as interaction keywords related to physical protein interactions, e.g. bind, phosphorylate. That would allow us to rank the results in a more nuanced way.

To reduce the curation load even more, we can use a text mining tool such as iHOP (45), which is able to identify protein interactions in the literature. For the present use case, iHOP only retrieves sentences about one abstract with an interaction between IL10 and IL32. This abstract is a TP and therefore its curation is associated to an overhead of 0%. The reduced number of results produced by iHOP in comparison to the co-occurrence method is probably due to the more narrow definition of interaction that is codified in the iHOP algorithm.

Depending on the curation time available we may decide for any of the three filtering strategies exposed, which produce different levels of overhead, taking into account that the filtering process itself requires time. For a pair of proteins with a large number of results iHOP might be the best choice while sentence co-occurrence might be better suited for less-studied pairs.

## Discussion

Although curation with insufficient resources and fixed deadlines is common, it is, understandably, not very frequently described in the published literature. Some systematic reviews and meta-analyses are instances of curation with insufficient resources. Such studies describe high recall, low precision situations in which the objective is to identify every relevant article about a topic even if many FPs need to be reviewed as well. Trade-offs in such cases can be nonetheless necessary because curators cannot review beyond a certain number of articles. Computational tools can be constructed that try to optimize the filtering process (46, 47).

The clinical setting is also a typical environment for curation with insufficient resources, especially for busy clinicians working in evidence-based medicine (48–50). Clinicians need to make decisions in a situation of competing time commitments, and possible urgency due to patients’ conditions, which leads to restrictions in the curation time spent seeking and reviewing relevant information to produce a complete picture of a patient’s condition, diagnosis, potential treatment plans or outlook.

Pharmacovigilance is another area in which prioritizing curation efforts is necessary (51). Reviewing the post-marketing safety surveillance data for popular drugs can be overwhelming for the institutions and companies that monitor these drugs. Signal detection can be used to prioritize the evidence with higher priority and increase the efficiency of the process (52).

Creating a pathway network or a systems biology model is often another case of insufficient curation resources. A curation project involving, e.g. a heavily studied pathway such as the Wnt signaling pathway is bound to be based on a set of prioritized information (53) rather than on an exhaustive analysis.

Finally, pharmaceutical companies need to be able to search, process and review large datasets at every stage of the drug pipeline, including in areas such as target discovery, lead optimization, toxicology, clinical pharmacology, regulatory submission and repurposing. In target discovery, for example, there is a need for low recall, moderate/high precision and high value results. In such settings, filtering strategies that can deal with multiple types of data can be utilized to pinpoint valuable items (54, 55) across a large space of potential leads.

## Conclusions

I have proposed and analyzed some variables and metrics related to value, efficiency and productivity that can be used in the realm of biocuration projects under the constraints of insufficient resources and incoming deadlines and with the goal of maximizing ROC. Throughout the exposition, the overhead metric was presented as a measure of curation efficiency and used to illustrate different ideas about curation.

From the analysis, I have suggested the need to consider the adoption of more than one filtering strategy or adjustable strategies in settings in which similar curation projects may appear at different times, rather than settling for just one strategy that might have the highest F-measure. Having a good ‘toolbox’ can help in dealing with curation projects under changing resource constraints. Of course, much can be learned during the process of curation that can help further develop and fine-tune filtering strategies. Filtering errors and biases learned during curation can be used to improve filtering (56).

Additionally, the analysis suggests that it may not be necessary to seek filtering strategies of very high quality because, in the settings here described, all items have to be in any case curated and, thus, small percentages of FPs can be tolerated. On the other hand, when filtering strategies are of moderate quality, small erosions in their performance can quickly impact the curation efficiency.

The curation projects here discussed have different properties from those of continuously curated databases, partially because they aim at ‘fixed targets,’ instead of moving targets. As emphasized in the discussion, filtering strategies are highly dependent on the curation resources available, and, thus, curation resources should be the driving factor in the design of filtering strategies.
